# Pregnancy and Childbirth in Women With Idiopathic Intracranial Hypertension

**DOI:** 10.7759/cureus.30420

**Published:** 2022-10-18

**Authors:** David R Hallan, Andrea C Lin, Cyril S Tankam, Dennis Madden, Elias Rizk

**Affiliations:** 1 Neurosurgery, Penn State Health Milton S. Hershey Medical Center, Hershey, USA

**Keywords:** obstetrics, childbirth, pregnancy, outcomes, idiopathic intracranial hypertension, pseudotumor cerebri, neurosurgery

## Abstract

Background

Idiopathic intracranial hypertension affects many women of childbearing age. However, the literature is sparse regarding pregnancy outcomes for these women. The goal of this study is to investigate the relationship between pregnancy outcomes in patients with a diagnosis of idiopathic intracranial hypertension.

Methodology

The TriNetX Research Network database was used to query 57 healthcare organizations for patients with idiopathic intracranial hypertension while pregnant (cohort 1) versus those who were pregnant without idiopathic intracranial hypertension (cohort 2). Cohorts were propensity-score matched for confounders related to pregnancy outcomes. The primary outcomes of interest were ectopic or molar pregnancy, cesarean section, abortion, preterm labor, depression, pre-eclampsia or eclampsia, and mortality. Chi-square analysis and logistic analysis were used on categorical variables.

Results

Ectopic/molar pregnancy was seen in 106 (1.75%) versus 117 (1.93%) (odds ratio (OR) 0.904, 95% confidence interval (CI) (0.694, 1.179), p = 0.4572) patients in cohorts 1 and 2, respectively. Cesarean section was seen in 785 (12.94%) versus 886 (14.59%) (OR 0.869, 95% CI (0.784, 0.964), p = 0.0078) patients, abortion in 536 (8.83%) versus 682 (11.24%) (OR 0.765, 95% CI (0.679, 0.862), p < 0.0001), preterm labor in 498 (8.206%) versus 668 (11.01%) (OR 0.723, 95% CI (0.640, 0.816), p < 0.0001), depression in 1,057 (17.42%) versus 1,061 (17.48%) (OR 0.995, 95% CI (0.906, 1.093), p = 0.9238), and pre-eclampsia/eclampsia in 501 (8.26%) versus 492 (8.11%) (OR 0.1.02, 95% CI (0.896, 1.161), p = 0.7657). Mortality was seen in 68 patients in cohort 1 versus 13 patients in cohort 2 (OR 5.279, 95% CI (2.913, 9.564), p < 0.0001).

Conclusions

This retrospective study examined pregnancy outcomes for pregnant women with a diagnosis of idiopathic intracranial hypertension. Women with idiopathic intracranial hypertension do not have an increase in rates of abortion, ectopic/molar pregnancy, cesarean section, preterm labor, or depression when compared to women without idiopathic intracranial hypertension. The mortality rate was higher in the idiopathic intracranial hypertension cohort, but still very low. This study demonstrates that pregnancy is generally well tolerated in the idiopathic intracranial hypertension population.

## Introduction

Idiopathic intracerebral hypertension (IIH), also known as benign intracranial hypertension or pseudotumor cerebri, is a condition of undefined etiology that causes a chronic elevation in the levels of intracranial pressure [1,2]. It is a condition that primarily affects obese women, particularly those who experienced a rapid increase in weight over a short period [3]. This condition primarily presents as chronic diffuse headaches, and patients can experience other symptoms of increased intracranial pressure, such as pulsatile tinnitus and retrobulbar pain, and, left untreated, some patients can develop vision loss [2].

IIH often affects women of childbearing age [4-10]. Of those with IIH, pregnancy has a reported prevalence of 2-12% [4,7,11]. Pregnancy can act as a crucible for IIH as it is often associated with rapid weight gain, increased vasodilation, increased cardiac output, increased blood volume, increased sodium and water retention, increased central venous pressure, and increased Valsalva maneuvers during labor, which, in turn, increase intracranial pressure [4,6,11]. Thus, pregnancy is a unique and high-stakes time that demands careful and effective diagnostic and therapeutic action to best manage the safety of both the mother and the fetus. Because current research is limited, and as there are no concrete guidelines to help physicians determine the treatment of IIH in pregnancy, it is necessary to understand associations between pregnancy and IIH [4-6,11]. This study seeks to determine if there is a correlation between IIH and pregnancy complications such as ectopic or molar pregnancy, cesarean section, abortion, preterm labor, depression, pre-eclampsia/eclampsia, and mortality to elucidate if pregnancy is safe for individuals with IIH.

## Materials and methods

This study was designed using a retrospective case-control study model using data obtained from the TriNetX research network. TriNetX is a globally federated health research network that provides access to the electronic health records of patients across many healthcare organizations. This report was run on a set of healthcare organizations grouped into a subnetwork called Research, which includes 57 healthcare organizations. The database was queried using the International Classification of Diseases, Tenth Revision, Clinical Modification (ICD-10) and Current Procedural Terminology codes (CPT). Because TriNetX is a federated database, an institutional review board waiver was granted for the use of this database. The use of this database was guided by data in the literature validating this database for similar projects, and the exact details of this research network have already been described [12-14].

The database was queried for patients with a diagnosis of IIH before or up to eight months after the diagnosis of pregnancy and compared against a cohort of pregnant patients without IIH. Cohorts were generated on March 24, 2022. Analysis was performed with several primary endpoints, namely, ectopic or molar pregnancy, cesarean section, abortion, preterm labor, depression, pre-eclampsia or eclampsia, and mortality.

Analysis was performed using propensity score-matched cohorts using a greedy-nearest-neighbor algorithm with a caliper of 0.1 pooled standard deviations. This was to adjust for hypothesized confounders on the relationship between IIH and pregnancy outcomes of interest. The medical information adjusted for included age at the date of pregnancy, sex, race, and comorbidities of hypertension, obesity, hypothyroidism, diabetes, asthma, migraine, epilepsy, prior pregnancy with an abortive outcome, nicotine dependence, coagulation defects, edema, chronic obstructive pulmonary disease, history of spontaneous abortion, thrombocytopenia, history of ectopic pregnancy, antiphospholipid syndrome, prior missed abortion, systemic lupus erythematous, prior primary inadequate contractions, and prior induced abortions. Hazard ratios were calculated using R’s survival package v3.2-3 and were validated by comparing the output to SAS version 9.4. (SAS Institute Inc. Cary, NC, USA). Chi-square analysis and logistic regression were performed on categorical variables.

## Results

Table 1 shows patient count before and after propensity score matching. After propensity matching, 6,069 patients were identified in each cohort.

**Table 1:** Patient count before and after propensity score matching.

Cohort	Patient count before matching	Patient count after matching
1: Pregnancy and idiopathic intracranial hypertension	6,336	6,069
2: Pregnancy and no idiopathic intracranial hypertension	28,986	6,069

Table 2 shows the baseline demographics and characteristics of each cohort before propensity score matching, and Table 3 shows the baseline demographics and characteristics after propensity score matching. After matching, the age at index was 28.6 ± 8.7 years and 28.6 ± 9.1 years for cohorts 1 and 2, respectively. Overall, 50.90% versus 51.20% of patients were white, 32.00% versus 32.40% were black or African American, and 16.00% versus 15.40% were of unknown race. Characteristics with less than 10 patients or without significant differences prior to matching were not included in Table 2 and Table 3.

**Table 2:** Baseline demographics and characteristics before propensity score matching.

Cohort	Code	Demographic/diagnosis	Mean ± SD	Patients	Percentage of cohort	P-value	Standard difference
1	AI	Age at index	28.5 ± 8.7	6,191	100%	<0.001	0.344
2			32.2 ± 12.6	26,276	100%		
1	2106-3	White		3,153	50.90%	0.223	0.017
2				13,156	50.10%		
1	2054-5	Black or African American		1,953	31.50%	<0.001	0.122
2				9,811	37.30%		
1	2131-1	Unknown race		1,020	16.50%	<0.001	0.201
2				2,554	9.70%		
1	2028-9	Asian		45	0.70%	<0.001	0.13
2				608	2.30%		
1	O00-O08	Pregnancy with an abortive outcome		358	5.80%	0.17	0.02
2				1,642	6.20%		
1	O36.80	Pregnancy with inconclusive fetal viability		159	2.60%	0.533	0.009
2				639	2.40%		
1	O03	Spontaneous abortion		164	2.60%	0.443	0.011
2				743	2.80%		
1	O00	Ectopic pregnancy		73	1.20%	0.013	0.037
2				423	1.60%		
1	O02.1	Missed abortion		74	1.20%	0.923	0.001
2				318	1.20%		
1	O20	Hemorrhage in early pregnancy		400	6.50%	<0.001	0.075
2				2,218	8.40%		
1	Z37.1	Single stillbirth		11	0.20%	0.01	0.031
2				18	0.10%		
1	I10-I16	Hypertensive diseases		1,129	18.20%	0.001	0.047
2				5,274	20.10%		
1	E65-E68	Overweight, obesity, and other hyperalimentation		1,986	32.10%	0.011	0.036
2				7,991	30.40%		
1	E03	Hypothyroidism		397	6.40%	0.003	0.043
2				1,972	7.50%		
1	E08-E13	Diabetes mellitus		387	6.30%	<0.001	0.192
2				3,077	11.70%		
1	Z87.891	Personal history of nicotine dependence		355	5.70%	0.791	0.004
2				1,484	5.60%		
1	F40-F48	Anxiety, dissociative, stress-related, somatoform, and other nonpsychotic mental disorders		1,415	22.90%	0.678	0.006
2				5,941	22.60%		
1	F30-F39	Mood (affective) disorders		1,351	21.80%	0.016	0.034
2				6,109	23.20%		
1	M32	Systemic lupus erythematosus		91	1.50%	0.235	0.016
2				336	1.30%		
1	D65-D69	Coagulation defects, purpura and other hemorrhagic conditions		333	5.40%	<0.001	0.051
2				1,124	4.30%		
1	O10-O16	Edema, proteinuria, and hypertensive disorders in pregnancy, childbirth, and the puerperium		656	10.60%	<0.001	0.184
2				1,469	5.60%		
1	F32	Depressive episodes		1,121	18.10%	0.004	0.041
2				5,184	19.70%		
1	F31	Bipolar disorder		263	4.20%	0.004	0.039
2				918	3.50%		
1	D68.61	Antiphospholipid syndrome		68	1.10%	0.012	0.033
2				204	0.80%		
1	D68.62	Lupus anticoagulant syndrome		56	0.90%	0.279	0.015
2				202	0.80%		
1	D69.6	Thrombocytopenia, unspecified		107	1.70%	0.156	0.02
2				527	2.00%		
1	G40	Epilepsy and recurrent seizures		247	4.00%	<0.001	0.117
2				527	2.00%		
1	G43	Migraine		1,498	24.20%	<0.001	0.351
2				2,896	11.00%		
1	G44	Other headache syndromes		1,815	29.30%	<0.001	0.308
2				4,340	16.50%		
1	J45	Asthma		1,003	16.20%	0.027	0.031
2				3,962	15.10%		
1	J44	Chronic obstructive pulmonary disease		67	1.10%	<0.001	0.108
2				659	2.50%		
1	O36.6	Maternal care for excessive fetal growth		15	0.20%	0.116	0.024
2				98	0.40%		
1	O36.0	Maternal care for rhesus isoimmunization		32	0.50%	0.014	0.032
2				82	0.30%		

**Table 3:** Baseline demographics and characteristics after propensity score matching.

Cohort	Code	Demographic/diagnosis	Mean ± SD	Patients	Percentage of cohort	P-value	Standard difference
1	AI	Age at index	28.6 ± 8.7	6,069	100%	0.792	0.005
2			28.6 ± 9.1	6,069	100%		
1	2106-3	White		3,091	50.90%	0.785	0.005
2				3,106	51.20%		
1	2054-5	Black or African American		1,941	32.00%	0.6	0.01
2				1,968	32.40%		
1	2131-1	Unknown race		972	16.00%	0.369	0.016
2				936	15.40%		
1	2028-9	Asian		45	0.70%	0.441	0.014
2				38	0.60%		
1	O00-O08	Pregnancy with an abortive outcome		350	5.80%	0.786	0.005
2				357	5.90%		
1	O36.80	Pregnancy with inconclusive fetal viability		156	2.60%	0.29	0.019
2				175	2.90%		
1	O03	Spontaneous abortion		160	2.60%	0.822	0.004
2				164	2.70%		
1	O00	Ectopic pregnancy		73	1.20%	0.672	0.008
2				68	1.10%		
1	O02.1	Missed abortion		74	1.20%	0.613	0.009
2				68	1.10%		
1	O20	Hemorrhage in early pregnancy		400	6.60%	0.971	0.001
2				401	6.60%		
1	Z37.1	Single stillbirth		11	0.20%	1	<0.001
2				11	0.20%		
1	I10-I16	Hypertensive diseases		1,088	17.90%	0.08	0.032
2				1,015	16.70%		
1	E65-E68	Overweight, obesity, and other hyperalimentation		1,936	31.90%	0.249	0.021
2				1,877	30.90%		
1	E03	Hypothyroidism		388	6.40%	0.882	0.003
2				384	6.30%		
1	E08-E13	Diabetes mellitus		384	6.30%	0.147	0.026
2				346	5.70%		
1	Z87.891	Personal history of nicotine dependence		343	5.70%	0.551	0.011
2				328	5.40%		
1	F40-F48	Anxiety, dissociative, stress-related, somatoform, and other nonpsychotic mental disorders		1,376	22.70%	0.069	0.033
2				1,293	21.30%		
1	F30-F39	Mood (affective) disorders		1,321	21.80%	0.388	0.016
2				1,282	21.10%		
1	M32	Systemic lupus erythematosus		86	1.40%	0.651	0.008
2				92	1.50%		
1	D65-D69	Coagulation defects, purpura, and other hemorrhagic conditions		303	5.00%	0.269	0.02
2				277	4.60%		
1	O09-O09	Supervision of high-risk pregnancy		1,213	20.00%	0.124	0.028
2				1,146	18.90%		
1	O10-O16	Edema, proteinuria, and hypertensive disorders in pregnancy, childbirth, and the puerperium		612	10.10%	0.329	0.018
2				580	9.60%		
1	F32	Depressive episodes		1,095	18.00%	0.104	0.03
2				1,027	16.90%		
1	F31	Bipolar disorder		260	4.30%	0.314	0.018
2				238	3.90%		
1	D68.61	Antiphospholipid syndrome		63	1.00%	0.303	0.019
2				52	0.90%		
1	D68.62	Lupus anticoagulant syndrome		55	0.90%	0.426	0.014
2				47	0.80%		
1	D69.6	Thrombocytopenia, unspecified		101	1.70%	0.888	0.003
2				103	1.70%		
1	G40	Epilepsy and recurrent seizures		223	3.70%	0.962	0.001
2				224	3.70%		
1	G43	Migraine		1,400	23.10%	0.948	0.001
2				1,397	23.00%		
1	G44	Other headache syndromes		1,725	28.40%	0.455	0.014
2				1,688	27.80%		
1	J45	Asthma		974	16.00%	0.486	0.013
2				946	15.60%		
1	J44	Chronic obstructive pulmonary disease		67	1.10%	0.931	0.002
2				66	1.10%		
1	O36.6	Maternal care for excessive fetal growth		15	0.20%	0.857	0.003
2				16	0.30%		
1	O36.0	Maternal care for rhesus isoimmunization		30	0.50%	0.266	0.02
2				22	0.40%		

Table 4 shows the outcomes after propensity matching. Ectopic/molar pregnancy was seen in 106 (1.75%) versus 117 (1.93%) (odds ratio (OR) 0.904, 95% confidence interval (CI) (0.694, 1.179), p = 0.4572) patients in cohorts 1 and 2, respectively. Cesarean section was seen in 785 (12.94%) versus 886 (14.59%) (OR 0.869, 95% CI (0.784, 0.964), p = 0.0078) patients, abortion in 536 (8.83%) versus 682 (11.24%) (OR 0.765, 95% CI (0.679, 0.862), p < 0.0001), preterm labor in 498 (8.206%) versus 668 (11.01%) (OR 0.723, 95% CI (0.640, 0.816), p < 0.0001), depression in 1,057 (17.42%) versus 1,061 (17.48%) (OR 0.995, 95% CI (0.906, 1.093), p = 0.9238), and pre-eclampsia/eclampsia in 501 (8.26%) versus 492 (8.11%) (OR 0.1.02, 95% CI (0.896, 1.161), p = 0.7657). Mortality was seen in 68 patients in cohort 1 versus 13 patients in cohort 2 (OR 5.279, 95% CI (2.913, 9.564), p < 0.0001).

**Table 4:** Outcomes after propensity score matching.

Outcome	Cohort 1, n (%)	Cohort 2, n (%)	Odds ratio (95% confidence interval)	P-value
Ectopic or molar pregnancy	106 (1.75)	117 (1.93)	0.904 (0.694, 1.179)	0.4572
Cesarean section	785 (12.94)	886 (14.59)	0.869 (0.784, 0.964)	0.0078
Abortion	536 (8.83)	682 (11.24)	0.765 (0.679, 0.862)	<0.0001
Preterm labor	498 (8.206)	668 (11.01)	0.723 (0.640, 0.816)	<0.0001
Depression	1,057 (17.42)	1,061 (17.48)	0.995 (0.906, 1.093)	0.9238
Pre-eclampsia/Eclampsia	501 (8.26)	492 (8.11)	1.02 (0.896, 1.161)	0.7657
Mortality	68 (1.12)	13 (0.214)	5.279 (2.913, 9.564)	<0.0001

## Discussion

The results of this study demonstrate that there is no significant increase in ectopic or molar pregnancy in patients with IIH. Likewise, it shows that cesarean section, abortion, preterm labor, depression, and pre-eclampsia/eclampsia rates are not higher in those with IIH versus those without IIH. However, the mortality rate was significantly higher, albeit low, in the IIH and pregnancy cohort versus those without IIH. Without individualized data, it is difficult to determine the cause of this increase in mortality. Being just above 1%, however, these results demonstrate that pregnancy is well tolerated in patients with IIH.

Although the literature is sparse regarding the management of IIH in pregnancy, several studies have made suggestions on how to best manage this patient population [4-6,11]. The literature has determined that therapeutic abortion is not indicated in this patient population, and the evidence from this study agrees [4,5,7,9,15]. Furthermore, vaginal delivery in IIH has not been associated with worse outcomes, although the mode of delivery is controversial [6,11]. Prolonged active labor has been shown to cause intracranial pressure to rise up to 71 mmHg; nevertheless, there is no evidence to suggest that a cesarean section is a better alternative [4,6]. Currently, the literature suggests that the recommendation for the mode of delivery be made per obstetrics [4]. A few studies have also looked at anesthesia during pregnancy and IIH. Reports show that there is no contraindication to spinal or epidural anesthesia [8,16]. Should a cesarean section and anesthesia be required, the goal is to avoid further increases in intracranial pressure, and regional anesthesia may be preferred [8,16].

While rapid weight gain is overall associated with IIH, and weight gain is common in pregnancy, a National Swedish case-control study of IIH demonstrated that risk factors for IIH do not include pregnancy [1,11,17]. Despite weight gain being a risk factor, it is not recommended for pregnant patients to lose or not gain weight. Rather, it is recommended that this patient population avoid excessive weight gain and strictly control their diets to avoid ketosis [4,5,9].

This study does not show an increase in depression among patients with IIH and pregnancy; however, prior literature has shown that IIH alone is associated with increased rates of anxiety and depression. Furthermore, the rates of depression and anxiety during pregnancy correlate with episodes of postpartum depression, and, therefore, it is important to focus on managing this as quickly as possible [4].

Our analysis was not without limitations. The major limitation of this study was that it was retrospective in nature. Furthermore, due to the nature of the database, we were unable to collect patient-level data on specific outcomes. We were unable to report on radiology information. We do not have information on the type of diagnostic test used for confirmation of the disease. We do not have data on fetal outcomes. Although propensity score matching was used for known confounders, unknown confounders may exist. In addition, some misidentification is inevitable in database studies.

## Conclusions

This retrospective study examined pregnancy outcomes for pregnant women with a diagnosis of IIH. It found that women with IIH do not have an increase in rates of abortion, ectopic/molar pregnancy, cesarean section, preterm labor, or depression than women without IIH. The mortality rate was higher in the IIH cohort, but still very low. This study demonstrates that pregnancy is generally well tolerated in the IIH population. Because of a lack of guidelines for its management, it is recommended that pregnant women with IIH do have multi-specialty expertise available.
